# Finding consensus on frailty assessment in acute care through Delphi method

**DOI:** 10.1136/bmjopen-2016-012904

**Published:** 2016-10-14

**Authors:** John T Y Soong, Alan J Poots, Derek Bell

**Affiliations:** NIHR CLAHRC Northwest London, Imperial College London, London, UK

**Keywords:** Frailty, Acute Care, Frailty Assessment, Older Persons

## Abstract

**Objective:**

We seek to address gaps in knowledge and agreement around optimal frailty assessment in the acute medical care setting. Frailty is a common term describing older persons who are at increased risk of developing multimorbidity, disability, institutionalisation and death. Consensus has not been reached on the practical implementation of this concept to assess clinically and manage older persons in the acute care setting.

**Design:**

Modified Delphi, via electronic questionnaire. Questions included ranking items that best recognise frailty, optimal timing, location and contextual elements of a successful tool. Intraclass correlation coefficients for overall levels of agreement, with consensus and stability tested by 2-way ANOVA with absolute agreement and Fisher's exact test.

**Participants:**

A panel of national experts (academics, front-line clinicians and specialist charities) were invited to electronic correspondence.

**Results:**

Variables reflecting accumulated deficit and high resource usage were perceived by participants as the most useful indicators of frailty in the acute care setting. The Acute Medical Unit and Care of the older Persons Ward were perceived as optimum settings for frailty assessment. ‘Clinically meaningful and relevant’, ‘simple (easy to use)’ and ‘accessible by multidisciplinary team’ were perceived as characteristics of a successful frailty assessment tool in the acute care setting. No agreement was reached on optimal timing, number of variables and organisational structures.

**Conclusions:**

This study is a first step in developing consensus for a clinically relevant frailty assessment model for the acute care setting, providing content validation and illuminating contextual requirements. Testing on clinical data sets is a research priority.

**Strengths and limitations of this study**

- Good participation rates from national experts of diverse disciplines.
- Triangulation of multiple methodologies to demonstrate agreement as well as stability.
- Research questions addressed are based on gaps of knowledge in literature.
- However, consensus does not necessarily mean correctness.
- Assessment models based on these findings will need evaluation and validation on clinical data sets.

## Introduction

Advances in public health, improved social care in developed countries and advances in clinical medicine have resulted in unprecedented acceleration of ageing of the world's population. In the UK, current estimates of life expectancy from birth are 82.9 years for women and 79.1 years for men,^1^ with 14.7 million persons aged 60 years or older, 3 million of which are aged 80 years or older.^2^

For some people, this progressive demographic shift is associated with a change in health profile, with increased number of comorbidities, functional dependence, social needs and healthcare complexity. In the UK, older patients use significant proportion of acute care services. Patients over the age of 65 constitute two-thirds of the acute and general hospital populations, accounting for 40% of all hospital bed days and 65% of National Health Service spend.^3^ Recent analysis suggests that population ageing may account for up to 40% of the increase in emergency admissions.^4^ Additionally, the acute care environment, characterised by high volume and time pressures, is particularly challenging for a frail patient, with National Clinical Audit of medical inpatient care suggesting poor overall assessment and management of clinical conditions common in older persons, namely delirium,^5^ falls^6^ and incontinence.^7^

Frailty is a commonly used term to describe older persons who are at increased risk for developing multimorbidity, disability, institutionalisation and death. There have been international efforts to reach agreement,^8–10^ but at present there is no absolute consensus on clinical and operational definitions for frailty.^11^ Researchers and clinicians agree that frailty's effects are multidimensional, its causes are multifactorial and that it results in increased vulnerability to external stressors. An agreement exists that frailty is intimately related to, but distinct from disability, vulnerability and multimorbidity.^12^ There is also general agreement that frailty as a concept is useful for identifying older persons at risk of adverse outcomes, and there is growing consensus that frailty (and its consequences) may be preventable.

However, consensus has yet to be achieved regarding the dimensions or variables that must be measured for an operational definition of frailty,^8^ or in fact how to best measure them,^13^ especially in the acute care setting. There is no agreement as to when and where these variables should be measured, the characteristics of a successful frailty assessment instrument and the organisational structures that will help facilitate it. Any existing frailty assessment tools have been developed for use in the community (eg, population studies; care homes) and validated for specific purposes (eg, predict admission to hospital; costing). Equally, specific measures of frailty are not routinely collected in clinical practice (eg, grip strength by calibrated dynamometer).

This study aims to address gaps in knowledge and agreement around frailty assessment in the acute medical care setting. Specific research questions include:

1. What can we measure routinely in clinical practice to aid recognition and improve care for frail patients?
2. When is the optimal time to assess frailty?
3. Where is the optimal place to assess frailty?
4. What characteristics are crucial for a successful frailty assessment tool?
5. Given time and volume pressures in acute care, what is the optimal number of frailty variables that can be reliably measured for each patient?
6. What organisational structures (ie, the manner in which people work to provide optimal care, not the physical setting) best facilitate frailty assessment and management?

## Methods

### Modified Delphi technique

The Delphi method was used initially by Dalkey and Helmer in the 1950s at the RAND Corporation in Los Angeles to forecast the result of potential Russian nuclear strikes on American defence capabilities.^14^ It has since been used in economic forecasting and policy as well as education research. Within the healthcare setting, it has been used widely to explore and gain consensus in diverse issues within primary care,^15^ mental health,^16^ medical education,^17^ nursing,^18^ other allied health professions,^19^ and policy and quality improvement.^20^

The premise of Delphi method stems from the underlying assumption that the consensus of a group of experts is more accurate than from individual experts. This consensus has utility where evidence is lacking or contradictory, thus precluding definitive conclusions. The Delphi method is a structured process for systematically collecting and aggregating informed judgements from experts on specific topics. It is an iterative technique characterised by repeated rounds of controlled feedback until a consensus is achieved, recognised by a termination criterion set in advance.

### Questionnaire development

A literature review focused on frailty assessments developed or validated in the acute care setting outlined variables associated with frailty that we coded into five groups: Social demographics, Phenotype model, High intensity service usage, Accumulated Deficits model, Bio-gerontological model (see online supplementary appendix 1). An electronic questionnaire was developed using Survey Monkey software. The electronic questionnaire was piloted to improve usability and validity. Wording and format changes occurred over two iterative cycles before the electronic survey was distributed to the expert panel, and changes were made when issues of clarity were raised by a separate panel of clinicians and qualitative researchers. Changes were on wording of questions, and not content, and were instigated if any single panellist raised a concern. A ‘trap question’ was included in the questionnaire as a consistency and measure of engagement, at the suggestion of a qualitative researcher during the iterative process. To ensure a high response rate by item, the electronic software was set up to require a response for key questions, and the survey length was minimised to optimise overall response.

10.1136/bmjopen-2016-012904.supp1 supplementary appendices

### Expert panel selection and recruitment

A formal stakeholder analysis identified individuals locally and regionally drawing on the network of this research unit. To be considered a stakeholder, people were either publishing in UK journals, or providing frailty care, or involved in charities pertaining to the topic. For the second round, formal stakeholder analysis was expanded to encompass national participants, using same criteria. All identified were invited to take part. The selection criteria meant that participants were academics, front-line clinicians and specialists from charities. The panellists were invited by email through Survey Monkey distribution software. There were two rounds and the overall participation rates for Round 1 and Round 2 were 72.7% (n=16) and 48.8% (n=41), respectively (see online supplementary appendix 2). Round 1 results were presented electronically within the Round 2 survey to all participants. Six participants completed Round 1 and Round 2, with two non-responders of Round 1 completing Round 2.

### Theory/calculation

Resultant data tables generated by electronic survey were exported to Microsoft Excel for analysis. Descriptive statistics described by Greatorex and Dexter^21^ were used to measure consensus and stability. SPSS v21 was used to calculate intraclass correlation coefficients for overall levels of agreement using two-way random ANOVA with absolute agreement and Fisher's exact test.

## Results

### Optimal frailty indicators in the acute care setting

**Figure 1:**
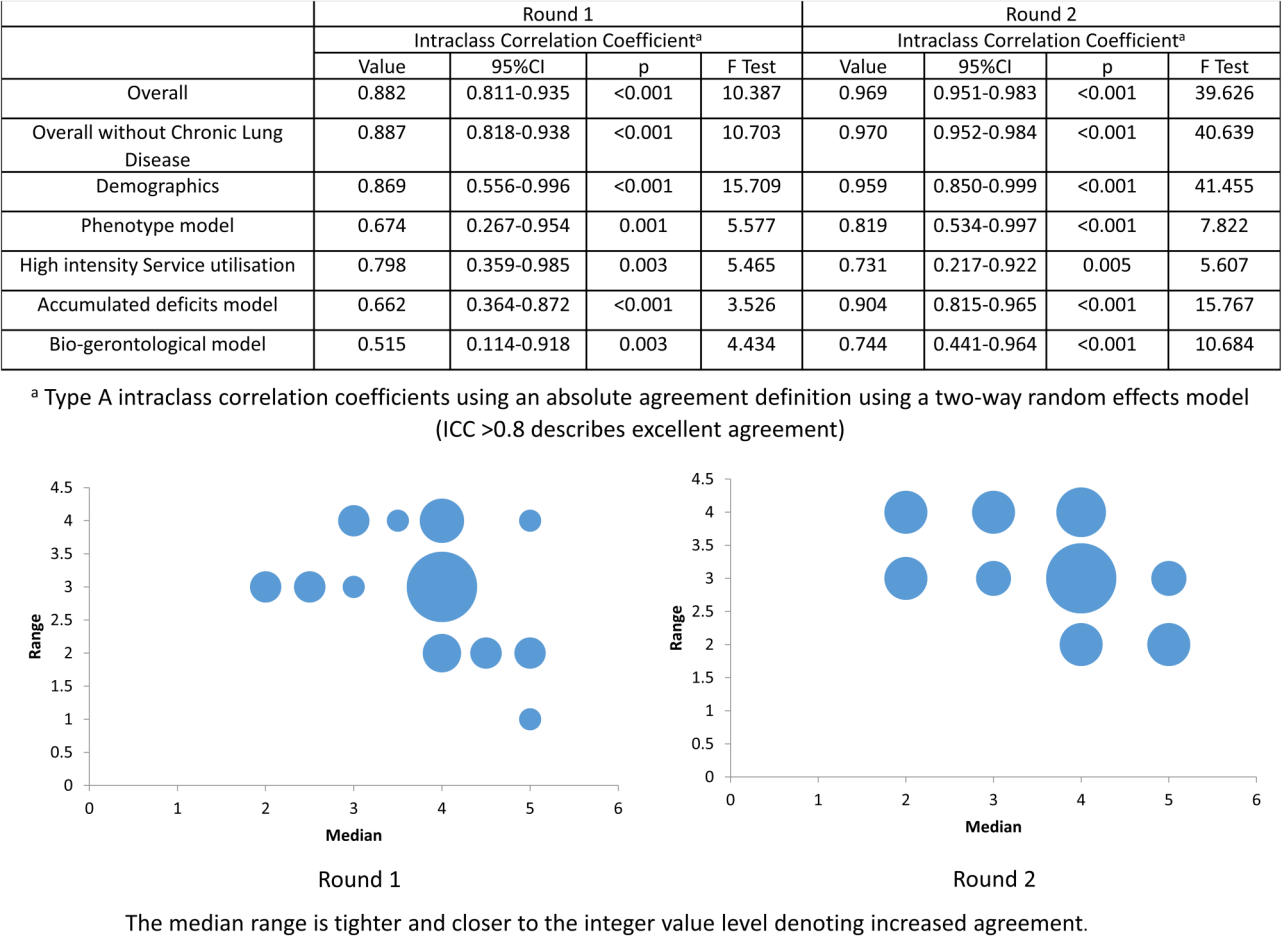
Summary of inter-rater reliability statistics for what frailty measures are useful in acute care.

Participants were asked to rate each of the 31 frailty indicators identified in the literature review (see online supplementary appendix 1) on a 5-point Likert Scale ranging from ‘Not useful at all’ to ‘Very useful’ for the best indicators of frailty in acute care. A threshold of >80% (‘useful’ or ‘very useful’) was taken as strong agreement by participants for usefulness and appropriateness in the acute care setting.^8^ A total of 38.7% (12 of 31, figure 1) indicators were finally accepted (table 1).

**Table 1:** List of frailty indicators agreed as most useful and appropriate for the acute care setting after the second round^1^

Frailty indicator	Classification	Per cent	N
Falls	Accumulated Deficits	95.1	39
Impaired cognition	Accumulated Deficits	95.1	39
Nutritional status	Accumulated Deficits	92.7	38
Functional dependence	Accumulated Deficits	90.2	37
Multiple morbidity	Accumulated Deficits	90.2	37
Impaired mobility	Accumulated Deficits	87.8	36
Multiple hospital admission episodes	High intensity service usage	87.8	36
Large package of care at home	High intensity service usage	85.4	35
Care home resident	High intensity service usage	82.9	34
Polypharmacy	Accumulated Deficits	82.9	34
Incontinence	Accumulated Deficits	80.5	33
Pressure ulcer risk	Accumulated Deficits	80.5	33

The Accumulated Deficits and High intensity service usage models were perceived by participants as most useful indicators of frailty in the acute care setting. The phenotype model was perceived as moderately useful and the bio-gerontological model was perceived as least useful. Patient demographics were perceived to have moderate or low usefulness (see online supplementary appendix 3).

Inferential consensus statistics show improved overall agreement between the rounds (figure 1). The modified fountain graphs demonstrate stabilisation of opinion and extent of agreement from Round 1 to Round 2. The ‘trap question’ displayed high levels of agreement between rounds, scoring as not useful.

### Optimal number of frailty indicators reliably measured in the acute care setting

**Figure 2:**
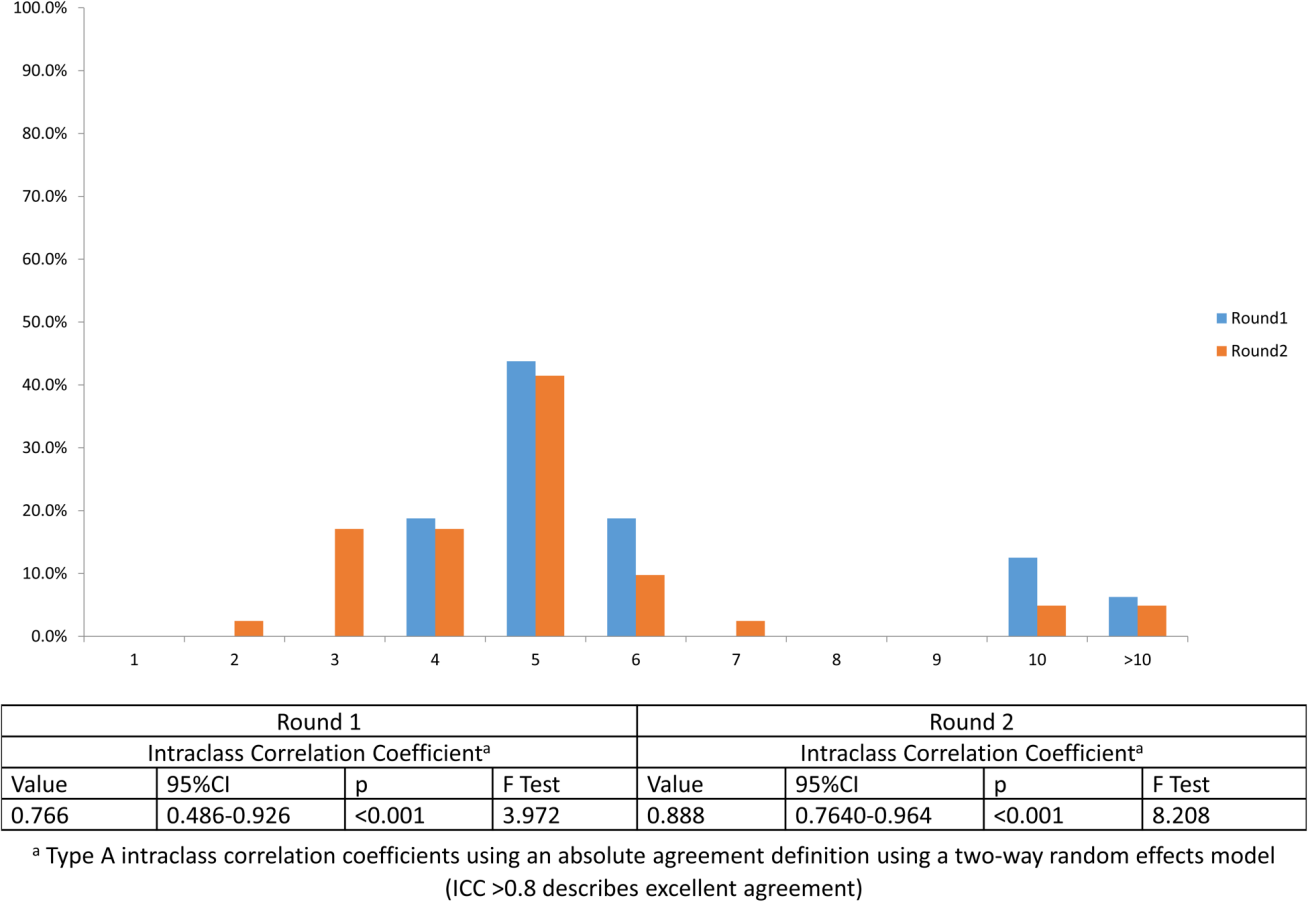
Number of frailty indicators reliably measured in the acute medical care setting.

Participants were asked ‘What is the maximum number of frailty indicators that can be reliably measured in acute care?’. Reliability was defined as always delivering care (100% compliance) with no variation in quality of care provided. A bimodal distribution was observed with a large mode of 5 in both rounds (43.8% and 41.5% in Round 1 and Round 2, respectively), and a smaller cluster of participants responding at the 10 and >10 item mark (18.8% and 9.8% in Round 1 and Round 2, respectively). This persistent clustering of data suggests true bipolarity of opinion,^22^ and was not taken as a marker of instability (figure 2).

### Optimal timing and setting to measure frailty in acute medical care

**Figure 3:**
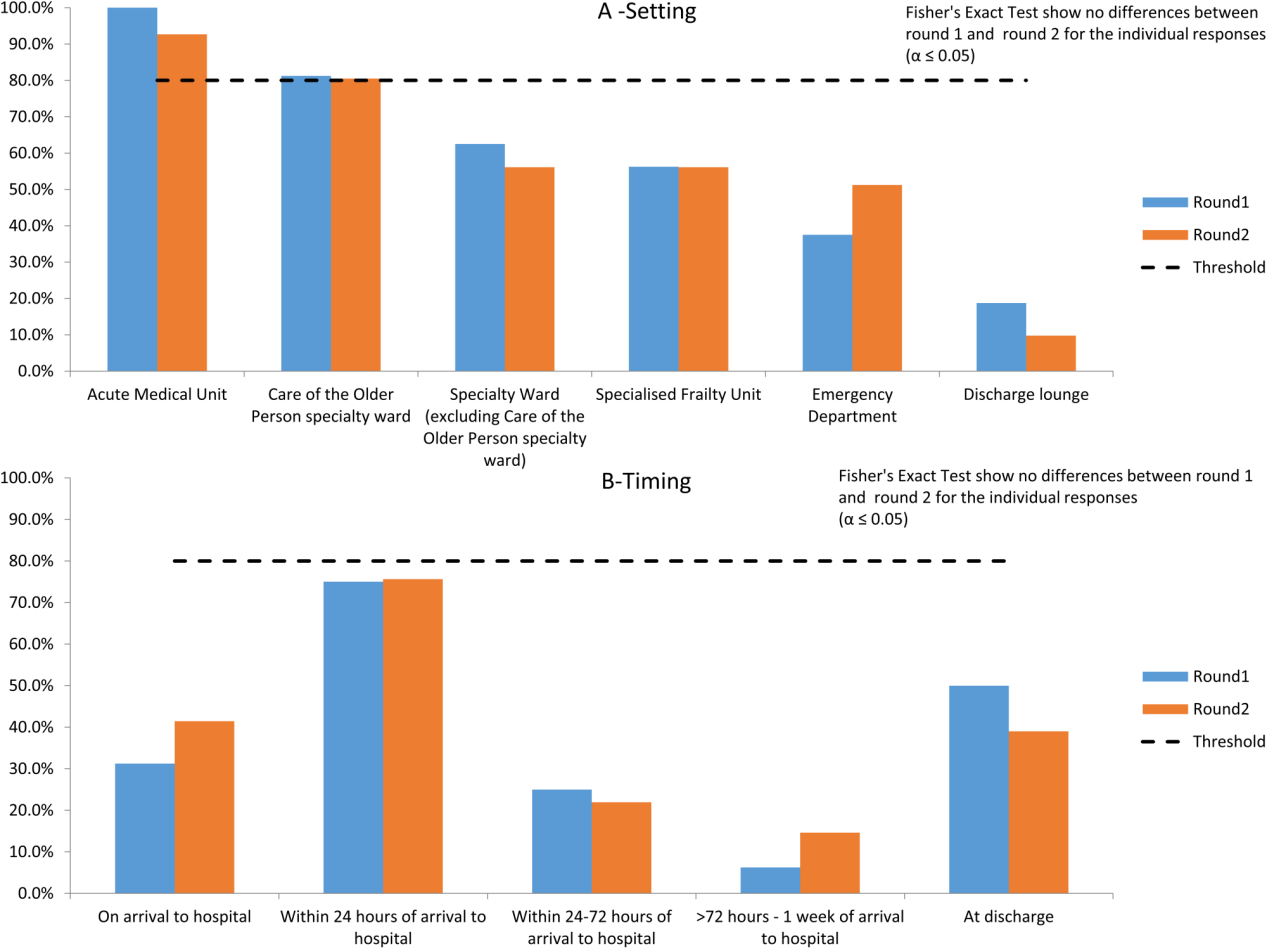
Optimal setting and timing of frailty assessment in acute medical care.

Participants were not forced to assign agreement to only one item, thus previously described inferential consensus statistics were not employed. A threshold of >80% was taken as agreement. Fisher's exact test, applied to ascertain if there was any difference in the proportions of individual response items between Round 1 and Round 2, was not significant at the α=0.05 level. The Acute Medical Unit (AMU; 100% and 92.7% in Round 1 and Round 2, respectively) and Care of the Older Person specialty ward (81.3% and 80.5% in Round 1 and Round 2, respectively) were perceived as participants as the optimal settings for frailty assessment acute care (figure 3A). Agreement was not achieved after two rounds for the optimal timing of frailty assessment in acute care, though ‘within 24 hours of arrival to hospital’ was the most frequently selected choice in both rounds (figure 3B).

### Optimal characteristics of a frailty assessment tool for acute medical care

**Figure 4:**
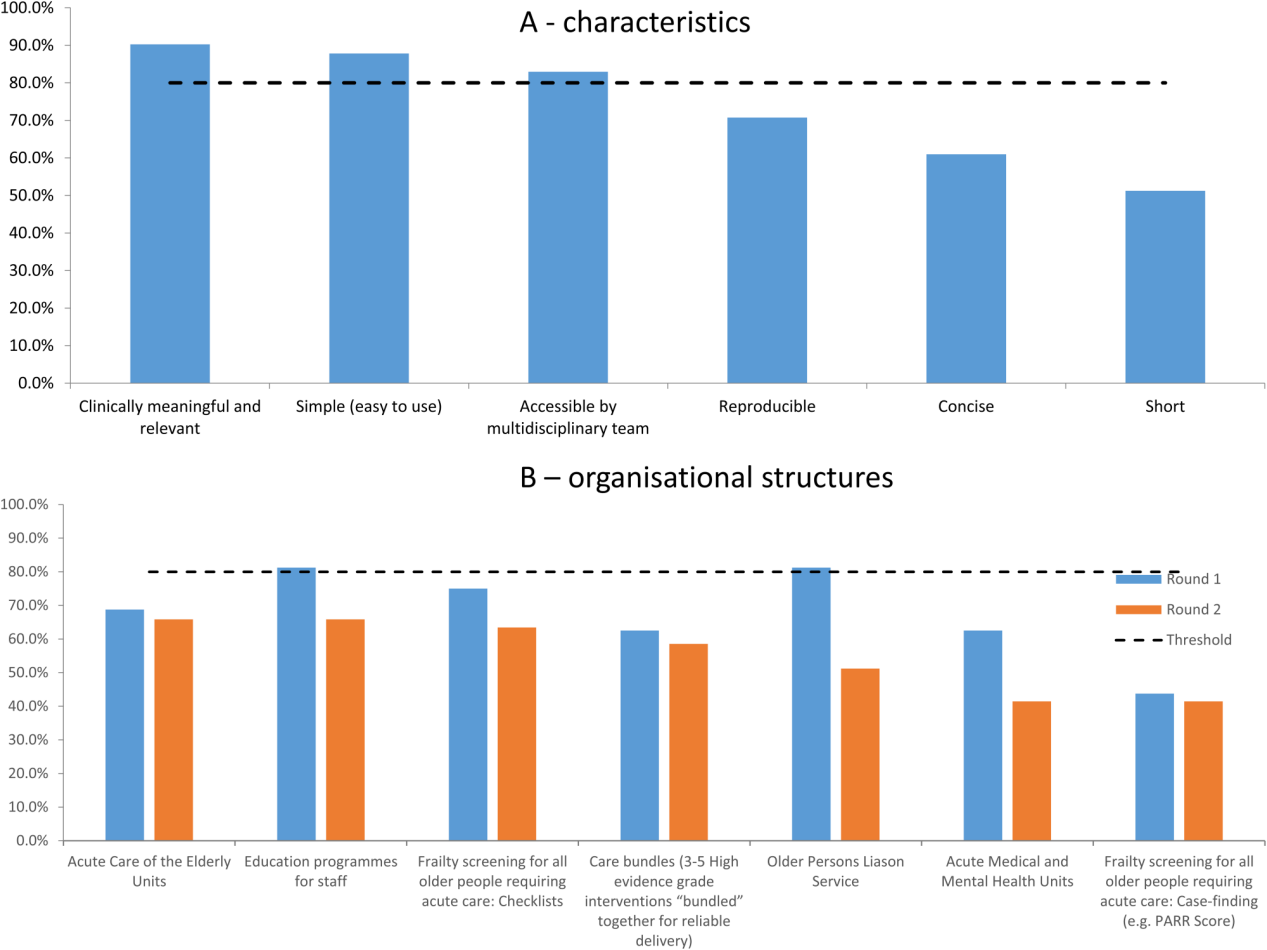
Optimal characteristics of a frailty assessment tool and effective organisational structures for frailty assessment in acute medical care.

Again, participants were not forced to assign agreement to only one item. In Round 1, panellists were asked an open question ‘What characteristics are crucial for a successful frailty assessment tool in acute care?’. The responses were coded by frequency and presented as choices in Round 2. The characteristics ‘clinically meaningful and relevant’, ‘simple (easy to use)’ and ‘accessible by multidisciplinary team’ were above threshold for agreement after the second round. Other comments include ‘open-ended responses’, ‘reproducible’, ‘short’, ‘avoids duplications’, ‘effective in picking up majority of cases’, ‘accurate and step wise with clear indication of next steps with regards to score thresholds’, ‘leads directly to enhanced care’, ‘low cost’, ‘Better at doing the job than ensuring that geriatricians (…)provide early and frequent senior review(…)’, ‘useful to person and lay carer’, ‘two separate scales may have to be used’ and ‘validated, calibrated and will inform practice’. There was also no consensus achieved regarding organisational structures that best facilitate frailty assessment by the end of Round 2 (figure 4A, B).

## Discussion

Diverse methods of Frailty measurement and operationalisation have roots in differing conceptual views regarding its definition. On one hand, frailty is seen as a subset of the elderly population with chronic inflammation, steroid hormone dysregulation, Vitamin D deficiency and sarcopenia as pathophysiological processes not fully elucidated, but culminating in a specific phenotype.^12^ ^23^ On the other hand, frailty is seen as the accumulation of biophysical deficit over time resulting in reduced resistance to external insults.^24^ These conceptual approaches are not mutually exclusive,^25^ and both add value to risk evaluation in the older person. Attempts to contextualise, combine or operationalise these concepts within the acute care setting have led to many different risk scoring systems (see online supplementary appendix 4). Generally, consensus is in favour of multidimensional measurement,^8^ ^10^ though unidimensional assessments (eg, grip strength or walking speed) have been applied.

This modified Delphi analysis suggests that measures of accumulated deficit and high intensity service usage are perceived as most useful and appropriate for quantifying frailty in the acute care setting. This may reflect the presence of many front-line clinicians as participants to this study, or that other measures of frailty were not collected within routine clinical practice in the acute care setting, or feasibly collected. This may not encapsulate current consensus definitions of frailty to its fullest extent,^8^ instead these findings represents an agreement of the most pertinent and applicable measures for the assessment and management of frailty in the acute setting. The frailty indicators agreed on in this study pertain to geriatric syndromes, which may be distinct from disability,^12^ as some are thought to be reversible. This is reflected in current national recommendations for quality of care for older persons with emergency and urgent care needs,^26^ ^27^ and in current clinical practice, with many of the perceived useful measures included within Comprehensive Geriatric Assessment,^28^ a resource intensive multidisciplinary assessment of patients deemed frail.

Participants perceived that the AMU and Care of the Older Person's ward were the optimal settings for frailty assessment, and that the frailty assessment tool should be clinically relevant, simple (easy to use) and accessible or useful to the multidisciplinary team. This may reflect the fact that the majority of emergency medical admissions occur through AMUs in the UK setting.^29^ There appeared to be clear bipolarity of agreement regarding the number of frailty indicators that could be reliably measured in the acute medical care setting, with the majority centred around 5 items (mode) and a smaller proportion for >10 items. This may reflect a difference in philosophy between screening and comprehensive assessment.

There was no consensus achieved for the optimal timing or organisational structures (these are working practices not a physicality, eg, Acute Frailty Unit or Acute Care of Elderly Unit) that best facilitates frailty assessment or management in the acute care setting, though ‘within 24 hours of arrival to hospital’ was consistently the most frequent choice. The lack of agreement regarding organisational structures may reflect differing contextual challenges and available resource for each participant. Thus, consensus may not ever be possible.

### Strengths

The Delphi method is an established mechanism for correlating informed judgement of a complex multidisciplinary topic into meaningful consensus, and for exploration of underlying reasoning or assumptions beneath these judgements.^30^ The Delphi method has several strengths. It is flexible with diverse applications for different aims and levels of resource, for example, modified Delphi,^31^ Policy Delphi^32^ and Real-time Delphi.^33^ It allows for preservation of anonymity to prevent intimidation or inhibition of opinion within potential hierarchical social structures and ensures that minority views are not eliminated early.^34^ It allows for participants to change their opinion between successive rounds with systematic refinement of consensus. Large groups of participants can be accommodated over large geographic areas. With increasing popularity of the electronic format, it is progressively time and cost efficient. Participant selection of experts promotes content validation, and allows networks to form.^35^ The strengths of this study also include good participation rates from national experts of diverse disciplines (reflecting the multidimensional nature of frailty), triangulation of multiple methodologies to demonstrate agreement as well as stability and clear research questions based on gaps of knowledge in literature.

### Limitations

The Delphi method has recognised limitations. The use of open questions in early rounds of the Delphi opens the study to researcher interpretation which risks potential bias.^36^ Attrition of participants in subsequent rounds can lead to sample bias.^37^ The selection of panel ‘experts’ has been challenged as subjective.^31^ There is lack of consensus on the optimum panel size or criteria for termination.^38^ Equally, the underlying principle of the Delphi method may not be true in all cases: Consensus does not necessarily mean correctness. Frailty assessment models built on these consensus findings will require robust evaluation and validation on clinical data sets.

In this study, specific limitations include a fairly low participant number, yet comprised of national experts, with a decline in response rate in the second round (although with an increase in number). Non-response consequently means only the views of those engaging in the process are determined.

In the literature review, we focused specifically on assessments that have been developed or validated within the acute care setting (see online supplementary appendix 4), which have been arguably primarily biomedical in nature. This strategy may have excluded useful predictor variables in frailty assessments used in the non-acute care setting.^39^ For example, the Tillburg Frailty Indicator^40^ is a validated instrument in the non-acute setting based on a predominantly biopsychosocial model, which explores social, psychological and environmental contributors to frailty. However, it is not clear how best to measure these parameters, nor how they affect overall outcomes, in the acute care setting.

## Conclusion

This study is a first step in developing a clinically relevant frailty assessment model for the acute medical care setting. It provides content validation for input variables into a model. It attempts to forecast the characteristics of a frailty assessment scale that may have high utility in the clinical setting, translating research into practice. Future work building and testing this model is a research priority.
