# Supplementary material for: Finding consensus on frailty assessment in acute care through Delphi method
Source: BMJ Open. 2016 Oct 14;6(10):e012904. doi: 10.1136/bmjopen-2016-012904 (PMC5073579; doi:10.1136/bmjopen-2016-012904)
Supplement: supplementary appendices [file bmjopen-2016-012904supp_appendices.pdf]

Appendix 1: Variables associated with frailty by model type

**Social demographics**	Age<br>Social Deprivation<br>Gender
**Phenotype model**	Unintended weight loss<br>Self-reported exhaustion<br>Weakness<br>Slow walking speed<br>Low physical activity
**High intensity service usage**	Recent hospital admission episodes<br>Multiple hospital admission episodes<br>Large package of care at home<br>Care home resident
*Geriatric syndromes<br><br>**Accumulated Deficits model**	Functional dependence*<br>Pressure ulcer risk*<br>Impaired cognition*<br>Incontinence*<br>Impaired mobility*<br>Falls*<br>Social isolation<br>Nutritional status<br>Multiple morbidity<br>Polypharmacy<br>Generalised anxiety and/or depression<br>Impaired vision<br>Impaired hearing
**Trap question**	Chronic lung disease
**Bio-gerontological model**	Chronic Inflammation (e.g. Raised serum IL-6)<br>Coagulopathy (e.g. Raised Factor VII/VIII levels)<br>Steroid hormone dysregulation (e.g. Low levels of DHEAS)<br>Low peripheral skeletal muscle bulk (e.g. peripheral quantitative computerized tomography (PQCT)<br>Low vitamin D levels

Appendix 2: Participants of the modified Delphi analysis

Round 1(n=16)			Round 2(n=41)		
**Position**	**Female**	**Male**	**Position**	**Female**	**Male**
Consultant Physician other	6.3%	12.5%	Academic	4.9%	2.4%
Clinical psychologist	0.0%	6.3%	Care Manager	2.4%	0.0%
Consultant Geriatrician	12.5%	25.0%	Chief Executive - Social Care	2.4%	0.0%
Consultant Psychiatrist	0.0%	6.3%	Consultant Geriatrician	12.2%	9.8%
Dietician	6.3%	0.0%	Consultant Physician other	7.3%	14.6%
Pharmacist	12.5%	6.3%	Dietician	2.4%	0.0%
Physiotherapist	0.0%	6.3%	GP	0.0%	2.4%
**Grand Total**	**37.5%**	**62.5%**	Nurse	4.9%	0.0%
			Pharmacist	4.9%	0.0%
			Physiotherapist	9.8%	2.4%
			Researcher	2.4%	2.4%
			Specialist Charity	0.0%	2.4%
			Specialist Trainee	7.3%	2.4%
			**Grand Total**	**61.0%**	**39.0%**

Appendix 3: Frailty indicators ranked by perceived utility in acute care by the end of round 2

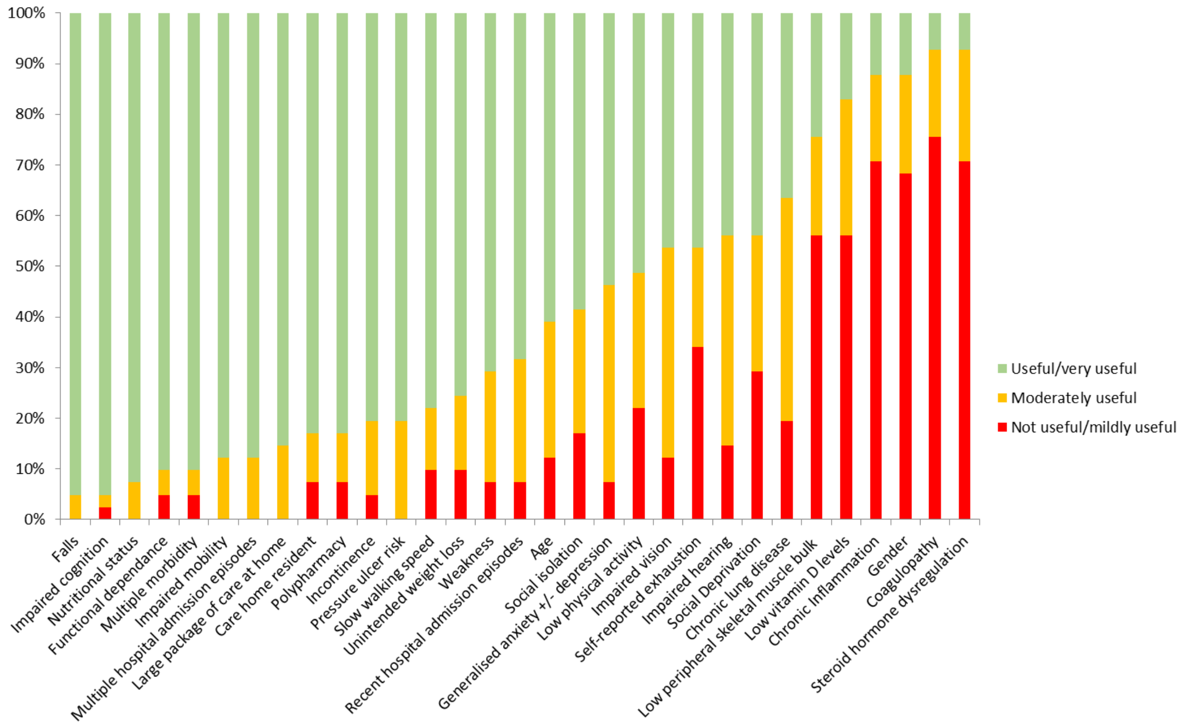

Appendix 4: Summary of frailty scores and criteria used within the acute care setting

Tool Name<br>(If applicable)	Frailty Criteria	Frailty Assessment Context	Description
NA	1. Cerebrovascular accident<br>2. Chronic and disabling illness<br>3. Confusion<br>4. Dependence in ADLs<br>5. Depression<br>6. Falls<br>7. Impaired mobility<br>8. Incontinence<br>9. Malnutrition<br>10. Polypharmacy<br>11. Pressure Sore<br>12. Prolonged bedrest<br>13. Restraints<br>14. Sensory impairment<br>15. Socioeconomic/family problems	Not Clear	Independent:<br>Independent in all ADLs with short term acute illness<br><br>Frail: meets any one of the criteria<br><br>Severely impaired:<br>Severe dementia and ADL dependence<br>Terminal illness
Identification of Seniors at Risk<br>(ISAR)	1. "Before the illness or injury that brought you to the emergency department, did you need someone to help you on a regular basis?" (Y/N)<br>2. "Since the illness or injury that brought you to the emergency department, have you needed more help than usual to take care of yourself?"(Y/N)<br>3. "Have you been hospitalized for one or more nights during the past 6 months (excluding a stay in the emergency department)?"(Y/N)<br>4. "In general, do you see well?"(Y/N)<br>5. "In general, do you have serious problems with your memory?"(Y/N)<br>6. "Do you take more than 2 different medications every day?"(Y/N)	On attendance to ED<br><br>Within 72 hours of admission on AMU	≥2 criteria
Hong Kong-Identification of Seniors at Risk<br>(HK-ISAR)	1. "Before the illness or injury that brought you to the emergency department, did you have any health problems that required you to limit your activities?"<br>2. " Have you visited a hospital emergency department during the past month? "<br>3. "Have you been hospitalised for one or more nights during the past 6 months? "<br>4. " Do you take more than two different medications every day? "<br>5. " In case of need, can you count on someone close to you? "<br>"Do you usually have enough income to meet your daily needs?"	On discharge home from ED	≥2
Triage Risk Screening Tool(TRST)<br>5-item<br><br><br><br><br><br>*6-item*	1. History or evidence of cognitive impairment (poor recall or not oriented)<br>2. Difficulty walking/transferring or recent falls<br>3. ≥ 5 medications<br>4. ED use in previous 30 days or hospitalization in previous 90 days<br>5. Emergency department nurse concern for elder abuse/neglect, substance abuse, medication noncompliance, problems meeting instrumental activities of daily living, or other<br>*6. lives alone/no caregiver*	On attendance to ED	≥ 2 criteria

Edmonton Frail Scale(EFS)	1. Cognition*<br>2. General Health Status*<br>3. Functional independence*<br>4. Social Support*<br>5. Medication use (≥5)<br>6. Nutrition<br>7. Mood<br>8. Continence<br>9. Functional performance<br>*Ordinal	a referral population for CGA in acute care wards, rehabilitation units, day hospitals and outpatient clinics	Not Frail 0-5<br>Apparently vulnerable 6-7<br>Mildly Frail 8-9<br>Moderately Frail 10-11<br>Severely Frail 12-17
Geriatric Syndromes	1. Presence of impaired cognition (MMSE≤23) at the time of assessment<br>2. Recurrent falls (two or more falls during the preceding 6 Months or three or more falls during thepreceding12 months)<br>3. A fall as a presenting complaint (cardiac/neurological causes of falls were not included, but unexplained non-accidental falls were included)<br>4. Impaired mobility at the time of assessment (requiring more than minimal assistance to stand up from a standard chair and walk 3 m)<br>5. Dependent in at least one PADL 2 weeks before admission (requiring assistance from another person)<br>6. Presence of urinary incontinence 2 weeks before admission (occasional stress incontinence was not considered sufficient).<br><br>Different models for different outcomes:<br>1. 90th percentile hospital length of stay<br>a. Lives alone<br>b. Distressed informal caregiver(s)<br>c. Impaired locomotion(admission)<br>d. Poor self-report health<br>e. Traumatic injury<br>2. Discharge to Higher level of Care<br>a. Impaired locomotion(admission)<br>b. Unstable condition<br>3. Any ED or hospital use within 28 days post index ED visit<br>a. Any premorbid ADL impairment<br>b. Expresses anhedonia<br>c. Any past ED visits(last 90 days)	Within 24 hours of admission<br><br>Not clear; During attendance to ED	≥1<br><br>NA
Mortality Risk Index(MRI)	1. Age ≥ 85(Y/N)<br>2. Dependent for ADL(Y/N)<br>3. Delirium(Y/N)<br>4. Malnutrition risk(Y/N)<br>5. Co-morbidity: Charlson Score 0-1<br>6. Charlson Score 2-4<br>7. Charlson Score ≥5	After admission, not clear	Weighted Score<br>≥ 2
Reported Edmonton Frailty Score(REFS)	1. Cognition*<br>2. General Health Status*<br>3. Functional independence*<br>4. Social Support*<br>5. Medication use (≥5)<br>6. Nutrition<br>7. Mood<br>8. Continence<br>9. Self-Reported performance<br><br>*Ordinal	After admission to medical and surgical wards – not clear	Not Frail 0-5<br>Vulnerable 6-7<br>Mild Frailty 8-9<br>Moderate Frailty 10-11<br>Severe Frailty 12-18

NA	1. Age ≥ 85(Y/N)<br>2. (Gender(M/F))*<br>3. Dependent for ADL(Y/N)<br>4. Delirium(Y/N)<br>5. Malnutrition risk(Y/N)<br>6. Co-morbidity: Charlson Score 0-1<br>7. Charlson Score 2-4<br>8. Charlson Score ≥5<br>9. Perceived health Scale(Duke Health Profile)-10pt increase<br>10. Number of children≥2±<br>11. Living alone[±]<br>*For mortality model<br>[±]For Nursing Home Placement	After admission, not clear	NA
Method for Assigning Priority Levels for Acute Care (MAPLe-AC)	1. ADL impairment cognitive impairment behavior<br>2. disturbance (verbally or physically abusive, socially inappropriate<br>3. behavior, resists care)<br>4. decline in decision<br>5. making<br>6. problems with medication management<br>7. pressure<br>8. ulcers<br>9. falls<br>10. problems with meal preparation<br>11. difficulty<br>12. swallowing	First 24 hours of hospital admission	Algorithm based interaction of criteria to form 5 levels of risk
Frailty Phenotype	1. Weakness: The maximum of the three grip strength using a dynamometer attempts was adjusted for gender and body mass index and lowest decile as cut off or self-reported<br>2. Slowness: Time to complete the 15-foot walk at normal pace was recorded and adjusted for gender and height using the same cutoffs for the lowest 20% or self-reported<br>3. Weight Loss: ''In the past year, have you lost more than 10 pounds unintentionally (i.e., not due to diet or exercise)?''<br>4. Exhaustion: ''Before the illness or injury that brought me to the ED, I felt that everything I did was an effort'' and ''Before the illness or injury that brought me to the ED, I felt that I could not get going''<br>5. Physical inactivity: by Rapid Assessment of Physical Activity (RAPA) scale	On discharge home from ED<br><br>Within 72 hours of admission on AMU<br><br>On discharge home from ED	≥3
The Study of Osteoporotic Fractures(SOF)<br>Frailty Index derived from the Study of Osteoporotic Fractures(FI-SOF)	1. weight loss (5% loss either intentional or unintentional over the last year)<br>2. self-report of low energy<br>3. low mobility (unable to rise from a chair five times)	Not clear; During their stay on Geriatric Evaluation and Management Unit (GEMU): a specialised ward designed to optimise a patient's chance of recovery following acute admission; Pre-selected for entry predominantly from Acute Medical Unit using the clinical judgement of geriatricians<br><br>Not clear;	SOF≥2

		During hospital admission on geriatric units<br><br>Within 72 hours of admission on AMU	
Fatigue, Resistance, Ambulation, Illness, Loss of Weight index (FRAIL),	1. fatigue (self-report)<br>2. resistance (unable to rise from a chair five times)<br>3. ambulation (slow walking speed)<br>4. illnesses (≥5 illnesses on Charlson's co-morbidity index (CCI)<br>5. loss of weight of 5% or more in the past year.	Not clear; During their stay on Geriatric Evaluation and Management Unit (GEMU):<br>a specialised ward designed to optimise a patient's chance of recovery following acute admission; Pre-selected for entry predominantly<br>from Acute Medical Unit using the clinical judgement<br>of geriatricians	FRAIL≥3
frailty index of cumulative deficits FI-CD	FI-CD<br>Help Bathing, Help Dressing, Help Transferring From a Bed to Chair and Back, Help Walking Around Home, Help Eating, Help Grooming, Help Toileting, Help Using Telephone, Help Shopping, Help Food Preparation, Help Housekeeping, Help Laundry, Help with Transportation, Help taking Medications, Help with Finances, Psychological Stress/Acute Disease in Last 3 Months, Previous Myocardial Infarction, Chronic Heart Failure, Peripheral Vascular Disease, Previous Stroke, Chronic Obstructive Pulmonary Disease, Renal Failure, Tumour, Diabetes, Orthostatic Hypotension, Pressure Sore or Skin Ulcer, Depression, Anxiety, Hearing Difficulty, Unable to Drive, Difficulty Chewing or Swallowing, Poor Dentition, Self-Reported Poor Health, Weight Loss > 4.5 kg in past year, Appetite, Self Report: "Everything is an effort", Self Report: "Could not get going", Low Physical Activity , Lives Alone, Low Community Mobility, Slow Walking Speed, Falls in Previous Year, Low Quality of Life, Mini Mental State Examination, Low Mid-Arm Circumference, Low Calf Circumference, Low Body Mass Index, Grip Strength, Low Protein Consumption, Self Reported Malnutrition<br><br>FI-CD:<br>32 items: difficulty with eating, dressing, walk around, getting in/<br>out bed, getting bath, toileting, using telephone, going out,<br>shopping, cooking, light house work, taking medicine, managing<br>money, arthritis, Parkinson's disease, glaucoma, diabetes, stomach<br>problems, history of heart attack, hypertension, history of stroke,<br>flu, broken hip, broken bones, trouble with bladder/bowels,<br>dementia, self-rated health, as well as problems with vision,<br>hearing, ear, teeth, and feet<br><br>FI	Not clear; During their stay on Geriatric Evaluation and Management Unit (GEMU):<br>a specialised ward designed to optimise a patient's chance of recovery following acute admission; Pre-selected for entry predominantly<br>from Acute Medical Unit using the clinical judgement<br>of geriatricians<br><br>Not clear;<br>During hospital admission on geriatric units<br><br>Within 72 hours of admission on AMU<br><br>First 24 hours of hospital admission	FI-CD>0.45<br><br>FI>0.25

	FI-AC: Not clear		
FI-CGA	55 variables (binary and ordinal) encompassing:<br>Cognitive Status, Delirium, Depression, Anxiety, Fatigue, Emotional Other, Motivation, Health Attitude, Speech, Hearing, Vision, Strength, Sleep, Daytime Drowsiness, Transfer, Walking, Aid, Balance, Falls, Falls Number, Bowel, Bladder, Weight, Appetite, Feeding, Bathing, Dressing, Toileting, Cooking, Cleaning, Shopping, Medication use, Driving, Banking, Medical problems, Count of medications	First 24 hours of hospital admission	FI<0.35 Not frail<br><br>FI-CGA 0.35-0.45 Mildly Frail<br>FI-CGA 0.46-0.55 Moderately Frail<br>FI-CGA>0.55 Severely Frail
Frailty Index based on Ten Domain Comprehensive Geriatric Assessment(FI-CGA-10)	Ordinal<br>1. Cognition (MMSE)<br>2. mood and motivation (GDS-15)<br>3. hearing or sight problem<br>4. mobility (6 m walk time)<br>5. balance (standing ability)<br>6. bowel function<br>7. bladder function<br>8. function<br>9. ADLs<br>10. IADLs<br>11. nutritional status (MNA)<br>12. social resources	Not clear; During their stay on Geriatric Evaluation and Management Unit (GEMU):<br>a specialised ward designed to optimise a patient's chance of recovery following acute admission; Pre-selected for entry predominantly from Acute Medical Unit using the clinical judgement of geriatricians<br><br>Not clear;<br>During hospital admission on geriatric units	FI-CGA-10>13
Score Hospitalier d'Evaluation du Risque de Perte d'Autonomie index (SHERPA);	1. falls in the previous year<br>2. MMSE (first 21 questions)<br>3. bad self-perceived health<br>4. Age<br>5. ADL	Not clear; During their stay on Geriatric Evaluation and Management Unit (GEMU):<br>a specialised ward designed to optimise a patient's chance of recovery following acute admission; Pre-selected for entry predominantly from Acute Medical Unit using the clinical judgement of geriatricians	SHERPA>6
Multidimensional Prognostic Index(MPI)	Ordinal:<br>1. Activities of Daily Living<br>2. Instrumental activities of daiy living<br>3. Short Portable Mental Status Questionnaire<br>4. Co-morbidity index<br>5. Mini Nutritional assessment<br>6. Exton Smith Scale/Braden skin assessment<br>7. Number of medications<br>8. Social Support Network	Not clear; During their stay on Geriatric Evaluation and Management Unit (GEMU):<br>a specialised ward designed to optimise a patient's chance of recovery following acute admission; Pre-selected for entry predominantly from Acute Medical Unit using the clinical judgement of geriatricians<br><br>During hospital admission on geriatric units	MPI>0.66

hospital admissions risk profile(HARP)	Ordinal<br>1. Age<br>2. MMSE-21<br>3. IADL	Not clear; During their stay on Geriatric Evaluation and Management Unit (GEMU):<br>a specialised ward designed to optimise a patient's chance of recovery following acute admission; Pre-selected for entry predominantly<br>from Acute Medical Unit using the clinical judgement<br>of geriatricians	HARP≥4
Adapted Katz index	Dependency for<br>1. feeding<br>2. washing<br>3. grooming<br>4. dressing<br>5. toileting<br>6. transferring from a bed or chair and walking	Not clear; During their stay on Geriatric Evaluation and Management Unit (GEMU):<br>a specialised ward designed to optimise a patient's chance of recovery following acute admission; Pre-selected for entry predominantly<br>from Acute Medical Unit using the clinical judgement<br>of geriatricians	Adapted Katz≥1
Lawton's instrumental Activities of Daily Living Scale	Dependency for<br>1. telephoning<br>2. shopping<br>3. food preparation<br>4. housekeeping<br>5. laundry<br>6. transport<br>7. medication<br>8. finances	Not clear; During their stay on Geriatric Evaluation and Management Unit (GEMU):<br>a specialised ward designed to optimise a patient's chance of recovery following acute admission; Pre-selected for entry predominantly<br>from Acute Medical Unit using the clinical judgement<br>of geriatricians	Lawton IADL≥3
Charlson Co-morbidity Index(CCI)	1. Myocardial Infarction<br>2. Congestive Heart Failure<br>3. Peripheral Vascular Disease<br>4. Cerebrovascular Disease<br>5. Dementia<br>6. COPD<br>7. Connective Tissue Disease<br>8. Peptic Ulcer Disease<br>9. Diabetes Mellitus (1 point uncomplicated, 2 points if end-organ damage)<br>10. Moderate to Severe Chronic Kidney Disease (2 points)<br>11. Hemiplegia (2 points)<br>12. Leukaemia (2 points)<br>13. Malignant Lymphoma (2 points)<br>14. Solid Tumour (2 points, 6 points if metastatic)<br>15. Liver Disease (1 point mild, 3 points if moderate to severe)<br>16. AIDS (6 points)	Not clear; During their stay on Geriatric Evaluation and Management Unit (GEMU):<br>a specialised ward designed to optimise a patient's chance of recovery following acute admission; Pre-selected for entry predominantly<br>from Acute Medical Unit using the clinical judgement<br>of geriatricians	CCI≥5
NA	Grip Strength	Not clear; During their stay on Geriatric	Grip strength<br><18kg women

		Evaluation and Management Unit (GEMU):<br>a specialised ward designed to optimise a patient's chance of recovery following acute admission; Pre-selected for entry predominantly<br>from Acute Medical Unit using the clinical judgement<br>of geriatricians	<30kg men
NA	Walking Speed	Not clear; During their stay on Geriatric Evaluation and Management Unit (GEMU):<br>a specialised ward designed to optimise a patient's chance of recovery following acute admission; Pre-selected for entry predominantly<br>from Acute Medical Unit using the clinical judgement<br>of geriatricians	Walking speed<br><30s to walk 6m
Ávila-Funes	Phenotype(see above) AND Cognitive impairment –<br>MMSE lowest 25%	Within 72 hours of admission on AMU	Ávila-Funes≥3
Rothman	1. Mobility-<br>Gait speed of >10 s to walk back and forth over a 10 foot course<br>2. Physical Activity-<br>Score <64 for men, <52 for women on the physical activity scale for the elderly<br>3. Nutritional Status<br>Weight loss > 10lbs in past year (intentional and unintentional)<br>4. Cognition<br>MMSE<24	Within 72 hours of admission on AMU	Rothman's≥2
Clinical Frailty Scale(CFS)	1 Very Fit – People who are robust, active, energetic<br>and motivated. These people commonly exercise<br>regularly. They are among the fittest for their age.<br>2 Well – People who have no active disease symptoms but are less fit than category 1. Often, they<br>exercise or are very active occasionally, e.g. seasonally.<br>3 Managing Well – People whose medical problems<br>are well controlled, but are not regularly active<br>beyond routine walking.<br>4 Vulnerable – While not dependent on others for<br>daily help, often symptoms limit activities. A common<br>complaint is being "slowed up", and/or being tired<br>during the day.<br>5 Mildly Frail – These people often have more	Within 72 hours of admission on AMU<br><br>Not clear; during inpatient stay on general internal medicine ward<br><br>Not clear; during inpatient stay under geriatricians based on geriatric targeting criteria that included delirium, deconditioning, functional impairment, gait<br>abnormality and falls, multiple medical diagnoses, and psychosocial problems	CFS>6

	evident slowing, and need help in high order IADLs<br>(finances, transportation, heavy housework, medications).<br>Typically, mild frailty progressively impairs shopping and walking outside alone, meal preparation<br>and housework.<br>6 Moderately Frail – People need help with all<br>outside activities and with keeping house. Inside, they<br>often have problems with stairs and need help with<br>bathing and might need minimal assistance (cuing,<br>standby) with dressing.<br>7 Severely Frail – Completely dependent for personal care, from whatever cause (physical or<br>cognitive). Even so, they seem stable and not at<br>high risk of dying (within ~ 6 months).<br>8 Very Severely Frail – Completely dependent,<br>approaching the end of life. Typically, they could<br>not recover even from a minor illness.<br>9. Terminally Ill - Approaching the end of life. This<br>category applies to people with a life expectancy<br><6 months, who are not otherwise evidently frail.		
Stable gait, Unstable gait, needed Help to walk or was Bedridden (SUHB scale)	SUHB* Corresponds to Clinical Frailty Scale<br>1. Walking unaided – steady<br>2. Walking unaided – unsteady<br>3. Walking with help<br>4. Bedridden	Not clear; during admission on AMU	SUHB – walking with help
skin integrity; problems eating; incontinence; confusion; evidence of falls; and sleep disturbance(SPICES)	1. skin integrity;<br>2. problems eating; incontinence;<br>3. confusion;<br>4. evidence of falls;<br>5. sleep disturbance	Within 24 hours of admission	≥2

NA:Not applicable; LoS: Length of stay(days); DRG(Diagnosis-related groups); Emergency Department(ED); NH: Nursing Home; LR: Likelihood Ratio; OR: Odds Ratios; ADLS: Activities of Daily Living; CGA: Comprehensive Geriatric Assessment, AMU: Acute Medical Unit; HR: Hazard Ratio, MMSE: Mini-mental State Examination
